# Plasticity as a developing trait: exploring the implications

**DOI:** 10.1186/1742-9994-12-S1-S4

**Published:** 2015-08-24

**Authors:** Marco Del Giudice

**Affiliations:** 1 Department of Psychology, University of New Mexico. Logan Hall, 2001 Redondo Dr. NE, Albuquerque, NM 87131, USA

**Keywords:** Development, differential susceptibility, parental effects, parent-offspring conflict, plasticity, predictive-adaptive responses, reaction norms

## Abstract

Individual differences in plasticity have been classically framed as genotype-by-environment interactions, with different genotypes showing different reaction norms in response to environmental conditions. However, research has shown that early experience can be a critical factor in shaping an individual's plasticity to later environmental factors. In other words, plasticity itself can be investigated as a developing trait that reflects the combined action of an individual's genes and previous interactions with the environment. In this paper I explore some implications of the idea that the early environment modulates long-term plasticity, with an emphasis on plasticity in behavioral traits. I begin by focusing on the mechanisms that mediate plasticity at the proximate level, and discussing the possibility that some traits may work as generalized mediators of plasticity by affecting the sensitivity of multiple phenol types across developmental contexts. I then tackle the complex problem of the evolution of reaction norms for plasticity. Next, I consider a number of potential implications for research on parental effects and phenotypic matching, and conclude by discussing how plasticity may become a target of evolutionary conflict between parents and offspring. In total, I aim to show how the idea of plasticity as a developing trait offers a rich source of questions and insights that may inform future research in this area.

## Introduction

Living organisms possess a remarkable ability to respond to environmental inputs with changes in form and function. Phenotypic adjustments may occur on many different timescales, from durable and sometimes irreversible changes—what most authors label *developmental plasticity*—to short-term, easily reversible responses variously labeled as *contextual plasticity *[1], *activational plasticity *[2], or *phenotypic flexibility *[3]. Everywhere on this continuum one finds remarkable individual variation; within a given species or population, some individuals respond to their environment with large phenotypic changes, whereas others are barely affected. Understanding the origin and meaning of individual differences in plasticity has important implications for the study of animal and human behavior [4-7].

**Figure 1:**
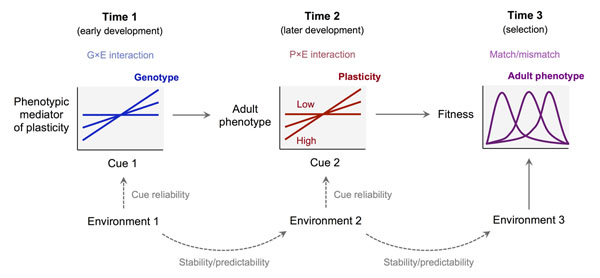
Plasticity as a developing trait. In this simplified scenario, plasticity at time 2 (i.e., the slope of the reaction norm at time 2) depends on the environmental conditions experienced at time 1. Specifically, plasticity at time 2 is mediated by a phenotypic trait that develops at time 1 through a genotype-by-environment (G×E) interaction. The adult phenotype develops at time 2 through a phenotype-by-environment (P×E) interaction. Finally, an individual's fitness is determined at time 3 by the match between an individual's phenotype and the state of the environment.

In the biological literature, developmental plasticity has been classically defined as the ability of a genotype to produce distinct phenotypes when exposed to different environments throughout ontogeny [8-10]. Consistent with this emphasis on genotypes, individual differences in plasticity are often framed as genotype-by-environment (G×E) interactions, with different genotypes showing different *reaction norms *to environmental conditions [11-13]. However, convergent findings have shown that early experience can be a critical factor in shaping an individual's plasticity to later environmental factors [6,7,14]. In other words, plasticity itself can be investigated as a developing trait that reflects the combined action of an individual's genes and previous transactions with the environment [7]. Individual differences in plasticity in response to later conditions can then be described as phenotype-by-environment (P×E) interactions, where the interacting phenotype is an individual's level of plasticity. In this perspective, the early development of plasticity is a critical step in the causal chain that connects the genotype to the realized adult phenotype. Figure 1 illustrates this concept in a simplified developmental scenario.

While the evolution of plasticity has been studied for decades [8,10,15], the concept of plasticity as a developing trait is still relatively new [4,6,7,14,16]. In this paper I explore some implications of the idea that plasticity is modulated by the early environment. In doing so, I focus specifically on long-term (developmental) plasticity in behavior, though most of the points I raise also apply to physiological and morphological development. I begin by focusing on the mechanisms that mediate plasticity at the proximate level, and discussing the possibility that some traits may work as generalized mediators of plasticity across traits and contexts. I then tackle the complex problem of the evolution of reaction norms for plasticity and present some preliminary simulation results. Next, I consider potential implications for research on parental effects and phenotypic matching, and conclude by noting how plasticity may become a target of evolutionary conflict between parents and offspring. My goal is not to provide a systematic analysis of these issues; rather, I aim to identify interesting questions and potentially fertile insights that may inform future research in this area.

## Phenotypic mediators of plasticity

In abstract terms, plasticity is a feature of an individual's reaction norm for a given trait; for example, linear reaction norms are characterized by two parameters (intercept and slope), with plasticity corresponding to the slope parameter (see Figure 1). In practice, the fact that some individuals are more susceptible to contextual factors must depend on differences in the proximate mechanisms involved in collecting input from the environment and translating it into phenotypic effects [14]. Here I will refer to these traits as *mediators* of plasticity at the proximate level. In principle, different traits of an organism might achieve plasticity through completely different mediators, without any common pathways or mechanisms. However, there are several reasons to predict that multiple traits will often share the same mediators. If so, plasticity should generalize across a variety of traits and contexts, with different individuals showing consistently high or low plasticity in multiple traits. (With linear reaction norms, this would translate into a pattern of correlations between the slopes of different traits [7]).

An initial reason to expect shared plasticity between traits is that different aspects of the environment may be assessed through the same sensory systems and pathways. As a consequence, individual differences in sensory thresholds and information processing have the potential to affect plasticity in multiple traits at once. Similar considerations apply to basic aspects of learning processes, such as an individual's sensitivity to reward and punishment. To the extent that behavioral traits develop and become established through learning, the working parameters of the neural machinery that supports learning can have broad-ranging effects on the plasticity of behavior. Importantly, individual differences in sensory and learning parameters can be expected to affect both developmental (long-term) and contextual (short-term) plasticity. This suggests that long-term and short-term plasticity may be correlated, so that individuals showing increased responsiveness to sensory stimulation, rewards, and so forth may be also more developmentally plastic in response to early experience. In the psychological literature, this hypothesis has been explored in the theory of *biological sensitivity to context *[4,5,17].

At a deeper level, life history theory suggests other reasons for the evolution of shared plasticity mechanisms. An organism's life history strategy results from the coordinated expression of phenotypic traits spanning multiple domains—from growth, metabolism, and fertility to aggression, mating, and parenting [18-21]. To enable plasticity in life history strategies, these traits must be developmentally regulated to produce coadapted responses to key dimensions of the environment such as mortality risk, resource availability, and predictability [22-25]. In many biological systems, the need to balance robustness and controllability with rapid, flexible adaptation to change favors the evolution of “bow-tie” architectures [26]. Bow ties are characterized by a small set of conserved core processes (the “knot”) that transfer resources and/or information between a multiplicity of inputs “fanning in” toward the core and outputs “fanning out” in various directions [26]. In total, the evolution of plasticity in life history strategies should favor phenotypic architectures in which multiple traits—morphological, physiological, and behavioral—show developmental plasticity in response to the same aspects of the environment, and in which coordinated plasticity is mediated by a core of shared regulatory mechanisms (see also [27]). This does not imply that all the traits involved in life history allocations should show the same amount of plasticity, as different traits may be more or less constrained because of physical limitations and biological trade-offs. Rather, such an architecture would result in a pattern of robust correlations between the slopes of different traits in response to the same environmental factors.

Consistent with this view, life history allocations in vertebrates are controlled by a relatively small network of conserved, interconnected endocrine pathways that include the insulin/insulin-like growth factor 1 (IGF-1) system, the hypothalamic-pituitary-adrenal (HPA) axis, the hypothalamic-pituitary-gonadal (HPG) axis, and the hypothalamic-pituitary-thyroid (HPT) axis [19,28-30]. Within this network, the HPA axis seems to play a central role in collecting and integrating information about the social and nonsocial environment from multiple sources, such as the amygdala and limbic structures in the brain, the immune system, and the insulin/IGF-1 system. For this reason, the reactivity of the stress response system (the integrated network that includes the HPA axis and the autonomic nervous system) is a natural candidate for the role of shared mediator of plasticity across traits and contexts [31-33].

In recent years, developmental research in humans has accumulated evidence that high levels of physiological stress reactivity (adrenocortical and autonomic) and negative affectivity (irritability, shyness, fearfulness, nervousness) predict increased plasticity in a broad range of traits including sociability, aggression, impulsivity, depressive symptoms, and maturation timing [4,6]. Genetic studies aiming to identify “plasticity alleles” typically converge on genes involved in serotonergic and dopaminergic pathways [4,6,34]; these pathways are critically implicated in processing rewards and punishments and show deep, bidirectional connections with one another and with the stress response system [31,33]. Other candidates showing associations with plasticity include genes involved in HPA signaling such as the corticotropin-releasing hormone receptor 1 gene (*CRHR1*) [35], as well as the brain-derived neurotrophic factor gene (*BDNF*) and the acetylcholine receptor gene, both of which regulate learning and neuronal growth [6]. Converging findings that emotional and physiological reactivity are associated with increased plasticity have been reported in studies of nonhuman primates, birds, and rodents [32,36], although the evidence from nonhuman species is considerably more sparse.

Taken together, these findings suggest that plasticity in different traits may be influenced by a relatively small number of physiological and behavioral mediators with generalized effects. Theoretical considerations on the structure of bow-tie architectures and life history trade-offs point to the hypothesis that core mediators of plasticity may be highly conserved within taxonomic groups; however, much more research is needed in this regard. An intriguing possibility is that developmental plasticity may be usefully described in hierarchical terms. At the highest levels in the hierarchy one would find generalized mediators—such as physiological stress reactivity and affective reactivity—that contribute to plasticity across a broad range of behavioral, physiological, and morphological traits, and ultimately mediate broad-band environmental effects on an organism's life history strategy. The intermediate levels would include traits that affect plasticity within narrower domains; possible examples are learning-related traits such as sensitivity to reward and punishment, or mating-related traits such as HPG axis reactivity. Finally, the lower levels of the hierarchy would include specialized mechanisms that contribute to mediate the plasticity of particular phenotypes. The plasticity of any given trait would then reflect the combined effect of generalized mediators, domain-specific mediators, and specialized mechanisms, with the relative weight of each source of plasticity varying in relation to the specific traits considered.

## The evolution of reaction norms for plasticity

If plasticity is not a fixed property of the genotype but a developing trait that responds to early environmental influences, it follows that the development of plasticity can be described by a reaction norm just like that of other traits (Figure 1). In the simplified scenario depicted in Figure 1, plasticity at time 2 emerges from the G×E interaction between an individual's genotype and the environment at time 1, while the adult phenotype is determined by the P×E interaction between plasticity and the environment at time 2. Reaction norms at time 1 describe how different genotypes respond to the early environment by producing different levels of plasticity at the next developmental stage.

While the idea that the early environment can affect plasticity is generally accepted [7,14], surprisingly little is known about the evolution of reaction norms for plasticity. Broadly speaking, natural selection tends to shape reaction norms so as to maximize expected fitness across environments. By definition, the ultimate phenotypic effect of a given level of plasticity critically depends on the environment that the organism will encounter in the future. Moreover, organisms typically assess the state of the environment through indirect cues that may be more or less reliable and predictive. In total, optimal reaction norms for plasticity must solve the following problem (see Figure 1): given the cues sampled at time 1, what level of plasticity maximizes the expected fitness of the phenotype that will develop in response to the cues sampled at time 2? The answer is likely to depend on a number of factors—for example the reliability of cues, the predictability of environmental states, and the shape of the fitness function for different combinations of phenotypes and environments.

I used a simple simulation model to find optimal reaction norms for plasticity in a simple scenario analogous to that depicted in Figure 1. In the model, the fitness of the adult phenotype depends on its match with the environmental state at time 3. Fitness is highest when the trait has the same value as the environment, and declines for higher and lower values of the trait. As a hypothetical example, consider a species in which the optimal level of aggression increases with population density. The adult phenotype (e.g., aggression) develops following a linear reaction norm, based on an environmental cue sampled at time 2 (e.g., the concentration of a pheromone that correlates with population density). The reaction norms of individuals with different levels of plasticity (i.e., different slopes) cross at an intermediate value of the environmental variable (as in Figure 1). In turn, the slope of the reaction norm at time 2 is determined by an environmental cue sampled at time 1 (e.g., exposure to maternal cortisol during gestation, which also correlates with population density). Environmental states are correlated over time, with larger auto correlations indicating higher stability. The goal of the simulation is to find optimal reaction norms for plasticity at different values of cue reliability and environmental stability (see Additional file 1 for details).

**Figure 2:**
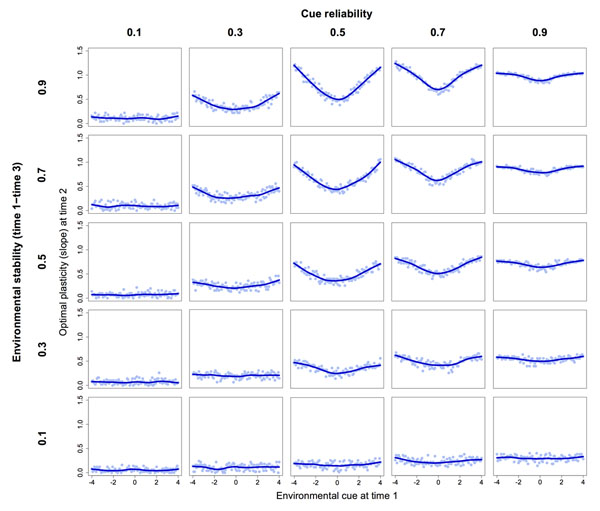
Optimal reaction norms for plasticity. In the simulation, the adult phenotype develops at time 2 through a crossover interaction between an environmental cue and the individual's plasticity. In turn, plasticity (i.e., the slope of the reaction norm) is determined at time 1 based on another environmental cue. Blue lines show the optimal slope at time 2 for different values of the cue received at time 1. Cue reliability is the correlation between a cue and the corresponding environmental state at each time point. Environmental stability is indexed by the autocorrelation between the state of the environment at time 1 and that at time 3. Fitness at time 3 is determined by a Gaussian function with the same characteristics across environmental states. Data represent 10,000 simulated individuals for each value of the environmental cue at time 1.

Simulation results are shown in Figure 2. All else being equal, higher levels of plasticity are favored when environmental cues are more reliable. Moreover, higher plasticity is favored in those regions of the environmental continuum where reaction norms at time 2 diverge. Since reaction norms at time 2 cross at the environmental midpoint, optimal reaction norms for plasticity will be U-shaped, with higher plasticity favored at both ends of the environmental continuum. This happens because environmental states are correlated over time; hence, the cue sampled at time 1 provides information about the likely state of the environment at time 3. If the cue sampled at time 2 is not perfectly reliable, the cue sampled at time 1 can be used to “correct” the information received at time 2; this is obtained by reducing plasticity if the cue sampled at time 1 is close to the point where reaction norms intersect.

Predictably, the curvature of the optimal reaction norm increases as the environment becomes more stable—that is, as early cues provide more information about the later state of the environment. Finally, the optimal curvature reaches a maximum for intermediate values of cue reliability. If reliability is very low, the cue sampled at time 1 contains little useful information; if on the other hand cues are extremely reliable, the information sampled at time 2 is so accurate that it does not require much adjustment. The exact shape of optimal reaction norms depends on the location of the crossover point at time 2; of course, if reaction norms at time 2 did not cross within the range of possible environmental states, the optimal reaction norms for plasticity would no longer be U-shaped but rather increase or decrease monotonically (as in the left or right half of the graphs shown in Figure 2). While these findings are clearly preliminary, the underlying logic is straightforward, and may point to a general pattern in the evolution of reaction norms for plasticity.

Intriguingly, these simulation results mirror a key prediction of the theory of biological sensitivity to context (BSC), an evolutionary-developmental theory of plasticity in humans [5,17]. According to BSC theory, higher levels of plasticity in behavior and physiology are favored in safe, supportive environments but also in stressful, harsh environments (i.e., at both ends of a continuum of ecological stress). This prediction is based on the hypothesized costs and benefits of plasticity in the face of social threats and opportunities, and has been supported in a number of empirical studies [4,17]. In light of the present results, the U-shaped reaction norm described by BSC theory might be explained more parsimoniously as a predictable outcome of plasticity evolution when (a) environmental conditions are at least moderately stable, (b) cues are at least moderately reliable (but not perfectly so), and (c) reaction norms with different levels of plasticity cross at intermediate values of the environmental variable. When these conditions apply, U-shaped reaction norms for plasticity may evolve to maximize phenotype-environment matching, regardless of the specific phenotypic and environmental variables involved. This insight has broad implications for studies of phenotypic development; notably, when the trait of interest is a putative mediator of plasticity (e.g., stress reactivity, negative affectivity) and there are reasons to expect a crossover P×E interaction in response to the later environment, the appropriate null hypothesis may be that of a curvilinear rather than linear reaction norm. Importantly, studies of human development find that interactions involving individual differences in plasticity often exhibit a crossover point at intermediate values of the environmental variable [6,12,34].

Of course, these initial results barely scratch the surface of the biological problem. Much more work is needed to characterize the evolution of reaction norms for plasticity under various ecological scenarios. Key questions concern the interplay between genetic and environmental factors in the early development of plasticity, and the selective processes that might maintain genetic variation in the potential for plasticity. Another interesting hypothesis is that plasticity developed early in life may not always have the function to promote accurate phenotype-environment matching later on. Instead, increased plasticity—especially in response to unreliable cues [37]—may sometimes work as a means to increase phenotypic diversity, for example in the context of “bet-hedging” strategies in unpredictably variable environments [37,38].

## Parental effects on plasticity

Developing organisms can use a variety of cues to gain information about the present and future state of the environment. The parental phenotype is a potentially rich source of predictive information, and can be adaptively used as a cue if environmental states are sufficiently correlated across generations [16,39]. Parental information may prove especially valuable for animals with complex brains and cognitive systems; as cognitive complexity increases, the parent behaves less like a passive transducer of the present environmental state and more like a sophisticated integrator of past and present information. Even more importantly, the parent can use its memory and sensory input to run a *predictive* model of the environment, in which current information is weighed in light of previous experience. For example, a pregnant mother may correctly predict that a certain negative state of the environment is going to be short-lived; as a result, she may avoid mounting a full-fledged stress response and sending an erroneous physiological signal to her offspring. In the literature on parental effects, the standard assumption is that early parental cues influence the offspring's future phenotype—for example large vs. small body size, early vs. late maturation—by bringing it closer to the optimum for the predicted state of the environment. When phenotypic matching later in life is regulated by early cues, many authors speak of a *predictive-adaptive response *[40].

The concept of plasticity as a developing trait suggests a subtle yet significant perspective shift on the logic of parental effects. In a nutshell, early parental cues may influence offspring development not (or not only) by directly shaping the target phenotype, but rather by modulating the offspring's level of *plasticity* to future environmental cues. In the scenario depicted in Figure 1, parental cues would be acting at time 1 to influence plasticity at time 2. The idea that early parental cues modulate plasticity is consistent with findings on the developmental effects of maternal hormones. Both in humans and nonhuman animals, it is often the case that exposure to maternal hormones—for example during pregnancy and lactation—has robust effects on traits known to mediate plasticity, such as negative affectivity and stress reactivity. For example, prenatal exposure to maternal stress and elevated glucocorticoids has been associated with negative affectivity and altered (typically increased) HPA reactivity in human infants and children; similar effects have been reported in other primates and rodents [41-44]. Two recent studies showed that glucocorticoid levels in mothers’ milk predict negative affectivity, both in human infants [45] and in Rhesus macaques [46]. Similarly, high levels of milk glucocorticoids have been found to predict increased HPA reactivity and anxiety in rodents [47]. Taken together, these findings suggest that mothers may be able to modulate their offspring's plasticity via hormonal cues during prenatal and early postnatal life, and that these effects may often involve generalized mediators of plasticity such as stress reactivity and negative affectivity.

**Figure 3:**
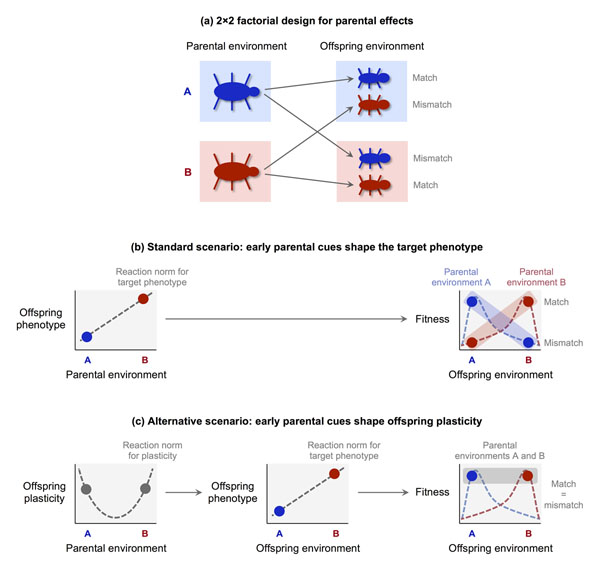
Parental effects on plasticity may change the predictions of experimental studies. (a) Schematic representation of a 2×2 factorial experiment on parental effects. (b) Experimental predictions based on the standard scenario, in which early parental cues directly shape the target phenotype. (c) Experimental predictions based on an alternative scenario, in which early parental cues shape plasticity to the later environment with a U-shaped reaction norm. In this scenario, the target phenotype (red/blue dots) is determined by the offspring environment rather than the parental environment. As a result, the expected fitness is the same for “matched” and “mismatched” phenotypes.

By modulating plasticity in a context-dependent manner, parental cues still provide indirect information about the likely state of the future environment. However, the implications can be remarkably different, particularly when parental effects are studied experimentally. In the standard experimental setup, parental effects are tested with a factorial design—often a 2×2 design in which the “treatments” represent two different states of the environment (Figure 3a). In the first phase of the experiment, parents are randomly assigned to one of the two environments; in the second phase, their offspring are also randomized between treatments, so that they may be reared in either a “matched” or “mismatched” environment (with respect to the one experienced by the parent). If parental cues directly affect the target phenotype, offspring reared in matched conditions are expected to perform better—that is, enjoy higher fitness—than those reared in mismatched conditions, resulting in a statistical interaction between parent and offspring treatments (Figure 3b; see [48]).

The predictions of factorial experiments may change dramatically if early parental cues do not directly shape the offspring's target phenotype, but rather modulate their plasticity to the later environment (Figure 3c). For example, if reaction norms for plasticity are U-shaped as in Figure 2, parental treatments of opposite sign may paradoxically end up having the *same* effect on offspring development—i.e., increasing the offspring's plasticity to later environmental conditions. Offspring of parents exposed to different treatments would then have similar levels of plasticity and would develop similar traits when raised in the same environment, regardless of whether the latter are “matched” or “mismatched” to the parental treatment. Accordingly, experimental results would show little or no interaction between parent and offspring treatments (Figure 3c). Detecting indirect parental effects of this kind would require three-step experimental designs in which the effect of the parental environment on the plasticity mediator (left panel of Figure 3c) is measured separately from the effect of the offspring environment on the target phenotype (center panel of Figure 3c). Moreover, if reaction norms for plasticity are U-shaped, experimental treatments should include at least three conditions (corresponding to high, medium, and low levels of the environmental variable) instead of the usual two (high vs. low).

Of course, parental effects on plasticity and parental effects on the target phenotype are not mutually exclusive, and may coexist to various degrees. However, the interaction effects detected in factorial experiments should become weaker and less reliable to the extent that early parental cues modulate plasticity rather than directly shape the adult phenotype. Interestingly, a recent meta-analysis of experimental studies in a wide range of species found that the overall support for the existence of adaptive parental effects is rather weak, and that when parental effects are reliably detected they tend to be very small [48]. Under the standard assumption that parental effects produce a specific phenotype matching a specific state of the environment, these findings are problematic; however, they may be explained in a different light if the primary effect of early parental cues is that of modulating offspring plasticity. While the apparent lack of strong parental effects does not by itself validate this alternative hypothesis, it is important to consider how the standard assumptions that guide experimental research may be revised in light of a more sophisticated understanding of developmental plasticity.

## Plasticity as a target of parent-offspring conflict

The idea that early parental cues contribute to shape the offspring's future plasticity raises the intriguing and still unexplored possibility that plasticity may sometimes become a major target of parent-offspring conflict. In sexually reproducing species, parents and offspring are not perfectly related; accordingly, their reproductive interests overlap only in part. Whenever a phenotypic trait is beneficial for the offspring but costly for the parent (or vice versa), the level of the trait that would maximize the parent's inclusive fitness is different from the level that would maximize fitness in the offspring. Both actors can increase their fitness by shifting the trait toward their own optimum, which creates the opportunity for conflict. This is the essence of *parent-offspring conflict theory *[49-52].

A common domain of parent-offspring conflict (POC) is food provision, which is costly for the parent and beneficial for the offspring. Offspring are selected to obtain more food than would be optimal for the parent to provide, while parents are selected to provide less food than would be optimal for the offspring. This basic conflict of interest may drive the evolution of elaborate systems of signaling and assessment, usually involving a degree of manipulation on both sides (e.g., exaggerated begging displays). The intensity of conflict—that is, the distance between the optima of parents and offspring—depends on several factors, such as the average relatedness among siblings and the characteristics of the mating system [50,52,53]. The logic of POC applies to a broad range of traits and behaviors including feeding, protection, sibling competition, offspring dispersal, patterns of growth and maturation, and even mating and reproductive decisions in both parents and offspring [51,52].

Parental effects are a natural arena for POC, as parents enjoy a unique opportunity to shape their offspring's phenotype in (partly) self-interested ways [54]. In turn, offspring face a complex decision problem; ignoring parental cues would protect them from manipulation, but also prevent them from acquiring vital information about the environment. This dilemma is especially acute in early development, when offspring typically lack the means to collect independent information from the environment; in those conditions, the benefits of the information transmitted by the parent may easily outweigh the costs of accepting some amount of parental manipulation. Note that this is not just a matter of parental cues being unreliable, since the distortions introduced by the parent are not random—rather, they are specifically directed *against* the offspring's best interests. Moreover, the information content of parental cues is not a stable quantity, as it can be expected to co evolve with the offspring's decision rules, leading to an evolutionary “arms race” between parents and offspring. Mathematical models indicate that the outcomes of conflict about parental effects critically depend on the cost of parental cues and the offspring's ability to discount or “filter” them [54]. When parental cues are costly to produce (as in the case of maternal hormones), the system is often expected to evolve toward a compromise, with the offspring's phenotype ending up somewhere in between the optimum of the offspring and that of the parent [54].

**Figure 4:**
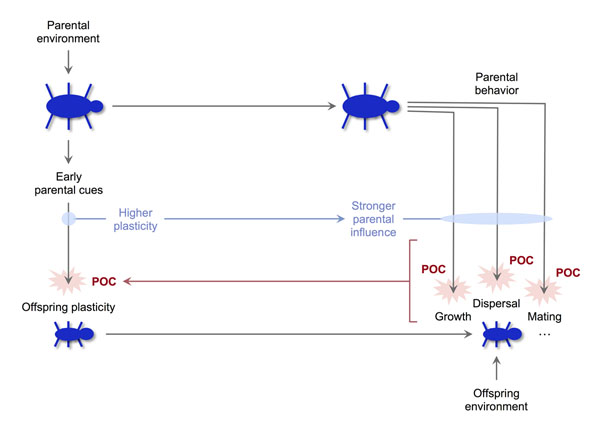
The logic of parent-offspring conflict about plasticity. Parents who increase offspring plasticity through early cues are able to exert a stronger influence on the development of multiple traits subject to parent-offspring conflict. As a result, parents are selected to increase offspring plasticity beyond the offspring's optimum, while offspring are selected to discount parental cues and develop lower levels of plasticity.

If, instead of directly affecting the target phenotype, parental cues primarily modulate offspring plasticity, then plasticity itself may become an important target of POC. This is most likely to occur if (a) parental effects on plasticity occur early in development; (b) parental cues affect generalized mediators of plasticity, such as stress reactivity; and (c) offspring spend considerable time in close interaction with their parents, for example in the context of extended parental care or cooperative breeding. (The last condition implies that parents represent an important aspect of the offspring's environment, and that parents and offspring have many opportunities to affect each other's fitness.) If these conditions are met, parents whose offspring are more plastic will be more able to influence their development in different areas and domains, including many that are potential targets of POC. In each of those domains, parents with more plastic offspring will enjoy a competitive advantage in manipulating their offspring in ways that maximize their own fitness (Figure 4). In other words, offspring plasticity may become a target of intense POC because of its *cumulative* effects on a wide range of narrower conflicts that will take place at later developmental stages (for example conflicts about parental investment, maturation, mate choice, and so on). All else being equal, parents will be selected to increase their offspring's plasticity (for example by increasing their early exposure to glucocorticoids), while offspring will be selected to “resist” parental cues and become less plastic than it would be optimal from the parents’ perspective [36]; this prediction holds even if parental cues transmit reliable information about the future state of the environment [54].

The idea that plasticity may become a target of POC offers a novel perspective on some important biological phenomena. As recognized in the literature on parental effects [48], POC about offspring traits will often shift the offspring's realized phenotype away from the value that would maximize fitness in a given environment. For this reason, the modest size of adaptive parental effects across species might reflect the widespread occurrence of POC in the development of offspring traits [48]. Parent-offspring conflicts about plasticity should increase the potential for mismatch because of the generalized effects of plasticity mediators. When a trait that mediates plasticity across domains (e.g., stress reactivity) is subject to POC, mismatches due to parental manipulation will spread to multiple traits at once, and their impact on offspring fitness will be magnified accordingly.

Moving to the level of physiological mechanisms, a conflict perspective may help make sense of the intricate dynamics of early parent-offspring interactions, including the reciprocal regulation of maternal and fetal hormones in mammals. In a recent paper, I suggested that POC about plasticity may explain some puzzling features of prenatal stress in humans [36]. For example, the placenta expresses a fetal enzyme that inactivates cortisol by converting it to cortisone; this mechanism is widely believed to serve a protective function, limiting fetal exposure to cortisol from maternal blood [43,55]. However, maternal tissues in contact with the placenta express another enzyme that converts cortisone to cortisol, apparently interfering with the protective role of the placenta [56,57]. This seemingly paradoxical finding might be explained by a conflict between mother and fetus about the optimal level of exposure to cortisol during prenatal development (see [36]).

## Conclusion

In this paper I discussed some implications of the idea that developmental plasticity can be shaped by the early environment. I reviewed initial evidence that some phenotypic traits—including affective reactivity and physiological reactivity to stress—may function as mediators of plasticity across contexts, and even across species. Convergence on a small number of generalized mediators of plasticity may enable coordination of multiple traits in service of an individual's life history strategy, and enhance the flexibility and robustness of the system by implementing a “bow-tie” architecture for developmental processes. In view of these considerations, I suggested that plasticity might be usefully described as a hierarchy of traits, from generalized mediators involved in the regulation of broad life history trade-offs to narrow mechanisms that mediate plasticity in specific domains or phenotypes. I then addressed the evolution of reaction norms for plasticity, and discussed some factors that may influence their shape. A simple simulation model suggested that crossover P×E interactions favor the evolution of U-shaped reaction norms for plasticity at moderate levels of environmental stability and cue reliability. In the following sections I explored the idea that early parental cues shape offspring plasticity to the later environment. In particular, I speculated that parental modulation of plasticity may contribute to explain the modest size of adaptive parental effects across species. Finally, I discussed how the development of plasticity may become a major target of parent-offspring conflict (with parents favoring higher levels of plasticity than offspring), and suggested that a conflict perspective on plasticity may help make sense of the intricate physiology of early parent-offspring interactions.

In total, I tried to show how the idea of plasticity as a developing trait has a remarkable potential to inform research on development and adaptation. The ramifications of this concept are not just theoretical—for example, I suggested that alternative views of plasticity may have substantial implications for the design and interpretation of experiments on parental effects. The insights discussed in this paper are tentative and largely speculative, and invite systematic investigation via formal modeling as well as empirical studies. Taken together, they suggest that our current understanding of developmental plasticity can be significantly expanded, and that many exciting findings may be waiting just beyond the horizon.

## Competing interests

The author declares no competing interests.

## Declarations

Publication costs for this article were funded by the German Research Foundation (FOR 1232) and the Open Access Publication Fund of Bielefeld and Muenster University.

## Supplementary Material

Additional file 1 **Simulation description** A description of the simulation discussed in the main text. Click here for file
