# Supplementary material for: Plasticity as a developing trait: exploring the implications
Source: Front Zool. 2015 Aug 24;12(Suppl 1):S4. doi: 10.1186/1742-9994-12-S1-S4 (PMC4722347; doi:10.1186/1742-9994-12-S1-S4)
Supplement: Additional file 1 — Simulation description A description of the simulation discussed in the main text. [file 1742-9994-12-S1-S4-S1.pdf]

Additional file 1: Del Giudice, M. ***Plasticity as a developing trait: exploring the implications.***

Simulation description

The goal of the simulation was to find optimal plasticity for different values of the environmental cue received at time 1 (C_1). The value of C_1 was varied from −4 to +4 in 100 steps of 0.08 each. Individual plasticity (P) was varied from 0 to 1.5 in 75 steps of 0.02 each. For each combination of C_1 and P, environmental states at time 1, 2, and 3, and environmental cues at time 2, were generated stochastically for 10,000 individuals as described by Eq. 1-4.

The true state of the environment at time 1 (E_1) was computed as

$$E_1 = r_C C_1 + X_1 \quad \text{(Eq. 1)}$$

where r_C is cue reliability and X_1 is a normally distributed random variable with mean = 0 and variance = 1. As a result, environmental states were also normally distributed, with mean = $r_C C_1$ and variance = 1.

The true environmental state at time 2 (E_2) was computed as

$$E_2 = \sqrt{r_E} E_1 + (1 - r_E) X_2 \quad \text{(Eq. 2)}$$

where r_E is a parameter quantifying environmental stability and X_2 is a normally distributed random variable with mean = 0 and variance = 1. This ensures that environmental states at time 2 also have variance = 1. Note that r_E is defined as the autocorrelation between environmental states at time 1 and time 3 (see Figure 2 in the main article); accordingly, the autocorrelation between E_1 and E_2 and that between E_2 and E_3 are both set to $\sqrt{r_E}$.

The environmental cue received at time 2 and the true environmental state at time 3 (E_3) were computed as

$$C_2 = r_C E_2 + (1 - {r_C}^2) X_3 \quad \text{(Eq. 3)}$$

and

$$E_3 = \sqrt{r_E} E_2 + (1 - r_E) X_4 \quad \text{(Eq. 4)}$$

where X_3 and X_4 are normally distributed with mean = 0 and variance = 1.

For each simulated individual, the adult phenotype (A) was determined by a crossover interaction between plasticity and the cue received at time 2 (C_2):

$$A = PC_2. \quad \text{(Eq. 5)}$$

The crossover point (i.e., the point at which reaction norms with different plasticity cross) corresponds to $C_2 = 0$.

Individual fitness (W) was computed with a Gaussian fitness function with mean = E_3 and standard deviation = 2, as follows:

$$W = \frac{1}{2\sqrt{2\pi}} e^{-\frac{(A-E_3)^2}{16}}. \quad \text{(Eq. 6)}$$

In Eq. 6, fitness is maximized when the adult phenotype matches the state of the environment at time 3 (i.e., when $A = E_3$). A standard deviation of 2 for the fitness function was chosen to ensure a gradual fitness decline over the range of simulated environmental states (Figure A1). However, the qualitative results of the simulation were not affected by the exact value of this parameter.

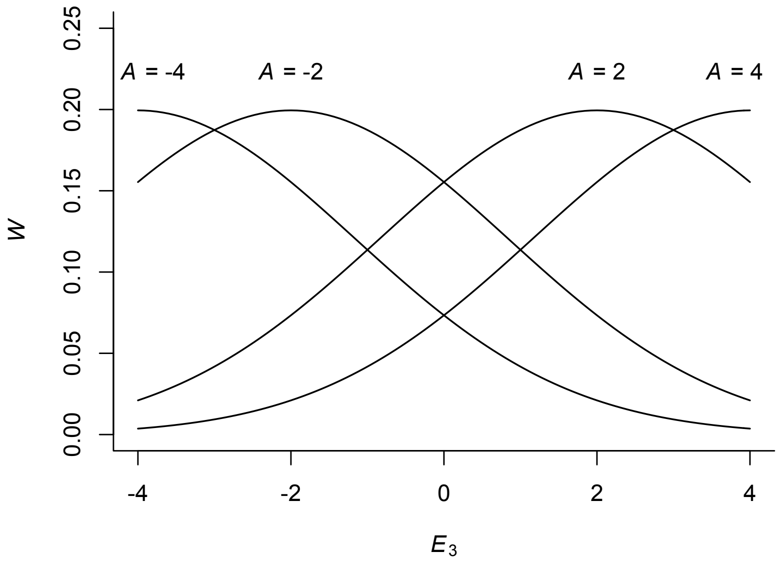

Figure A1. Fitness function for different values of the adult phenotype. A = adult phenotype; E_3 = environmental state at time 3; W = fitness.

For each combination of C_1 and P, expected fitness was computed as the average fitness ($\bar{W}$) of the 10,000 simulated individuals. Finally, the optimal level of plasticity (P*) was determined for each value of C_1 as the value of P with the maximum expected fitness.

The simulation was performed in R™ 2.15 (R Core Team, 2012. *R: A language and environment for statistical computing*. R Foundation for Statistical Computing, Vienna, Austria).
